# Larval density drives thermogenesis and affects microbiota and substrate properties in black soldier fly trials

**DOI:** 10.1016/j.isci.2025.112794

**Published:** 2025-05-30

**Authors:** Thomas Klammsteiner, Carina D. Heussler, Heribert Insam, Birgit C. Schlick-Steiner, Florian M. Steiner

**Affiliations:** 1 Universität Innsbruck, Department of Ecology, Technikerstrasse 25, 6020 Innsbruck, Tyrol, Austria; 2 BioTreat GmbH, Technikerstrasse 21, 6020 Innsbruck, Tyrol, Austria

**Keywords:** Entomology, Microbiome, Biotechnology

## Abstract

Industrial insect farming has potential for converting low-value organic waste into nutrient-rich insect biomass, producing valuable by-products like organic fertilizers. However, a better understanding of density-related thermogenesis and microbial dynamics is needed to enhance standardization and bridge gaps between laboratory and industry needs. This lab-scale study focuses on the black soldier fly (*Hermetia illucens*), which exhibits thermogenesis that intensifies with larval population size and its natural crowding behavior. Using high-resolution temperature monitoring and biomolecular methods, we found that doubling larval density (0, 1.25, 2.5, and 5 larvae/cm^2^) increased temperatures by 0.6°C–2.4°C, depending on the treatment. Adding potassium sorbate altered microbial profiles, increasing *Enterobacter* and decreasing *Providencia*, while promoting lactic acid bacteria. Density also impacted pH, water content, dry matter, volatile solids, and ash in the substrate. Our findings provide essential insights into managing microbial and thermal dynamics, offering valuable information for optimizing and standardizing conditions in rearing trials.

## Highlights

• Larval density drives thermogenesis and affects larval development
• Preserving agent alters larval and microbial activity
• Substrate properties and microbial composition depend on larval density

## Graphical abstract

**Figure:**
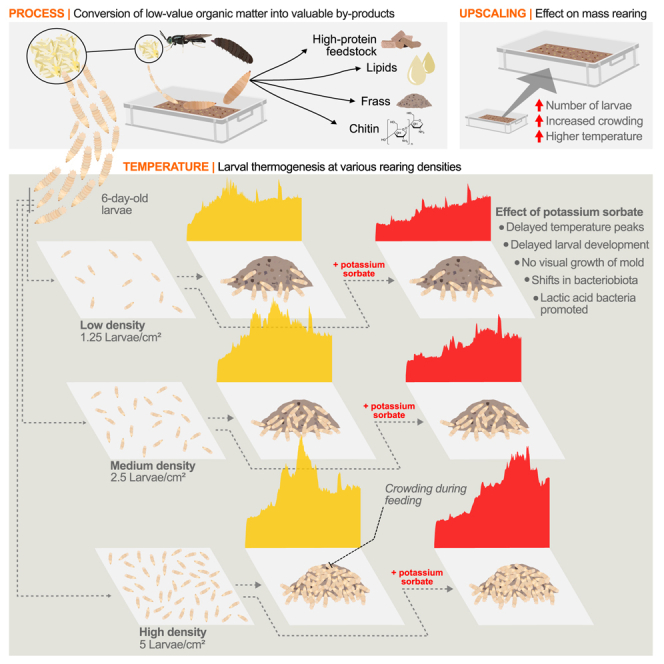

## Introduction

The use of insects for the bioconversion of organic waste into high-quality fat and protein provides a promising alternative to fishmeal and soy-based products for producing animal feedstuff.^1^^,^^2^ Over the last decade, mass rearing of insects has started to exploit this opportunity and has led to a global surge in large-scale insect farming ventures.^3^ Among the few insect species explored for mass rearing, the black soldier fly (BSF; *Hermetia illucens*) has proven to be ideal for this purpose. Its nutrient-rich larvae (BSFL) can digest a wide variety of organic wastes while at the same time producing a valuable organic fertilizer.^4^^,^^5^ The suitability of BSFL for high-density cultivation further enables the production of significant larval biomass, which can be enhanced by employing vertical farming techniques. Together, these attributes allow for environmentally and resource-efficient production, making the BSF a pivotal player in insect-based feed production.^6^

The emergence of an increasing number of large-scale facilities capable of processing tons of organic waste per day relies on stable processing cycles. However, the rearing process is a complex network defined by a wide range of physical, chemical, and (micro-)biological parameters.^7^^,^^8^ Along these lines, it is not only crucial to pay attention to maintaining appropriate rearing conditions but also to the thermogenesis of BSFL.^9^ Larval thermogenesis is increasingly recognized for its significant impact on productivity and the comparability of results in both academic research and industrial production.^9^ While most studies have focused on optimizing environmental temperatures to enhance BSF life-history traits,^10^^,^^11^^,^^12^^,^^13^ substrate temperature profiles driven by larval activity have been largely overlooked. In nature, the black soldier fly (BSF) is a frequent colonizer of carcasses.^11^ Accumulating maggot masses can induce local thermogenesis, while bacterial degradation processes raise overall carcass temperatures, with both larvae and bacteria influencing each other’s activity.^14^ Temperature, population size, and rearing density are intertwined parameters that can have wide ranging consequences for the biological and economic output.^15^ Larval densities are heterogenous across studies and in some cases not clearly reported, but efforts for establishing standards have been made.^16^

Recently, more studies addressed the issue of larval thermogenesis and started tracking substrate temperatures.^9^^,^^17^^,^^18^^,^^19^^,^^20^ For example, Jiang et al.^18^ reported peak temperatures of 50°C during larval bioconversion of food waste. Given that the upper thermal limit for BSF larvae is estimated to be 44°C, and constant exposure to environmental temperatures of 36°C–37°C already result in negative effects ranging from lower adult emergence and severe malformations^10^ to a significant reduction of survival from larva to adult,^13^ peak temperatures reaching 50°C could have lasting detrimental effects on colony fitness. In biological degradation processes, such as composting, microbial activity drives the heating process.^21^ The rearing substrate contains an abundance of microorganisms, all of which help degrade organic matter.^5^ In most studies, the monitoring of substrate temperatures was done sporadically but not continuously, using different methods, such as thermometers,^9^^,^^18^^,^^20^ temperature probes,^19^ or thermal-imaging cameras.^9^^,^^17^

This study aimed to understand how these density-driven temperature patterns complicate the comparability of experimental results. Specifically, it explores how varying larval densities influence BSLF thermogenesis and its effects on larval development, microbial communities, and substrate properties to improve experimental consistency and optimizing rearing conditions. We investigated the interplay between three larval densities (1.25, 2.5, and 5 larvae/cm^2^) and thermogenesis, assessing their combined impact on larval development and bacterial communities. We continuously monitored substrate temperatures and employed high-throughput amplicon sequencing, using larval population sizes typical for lab-scale experiments. To reduce the potential contribution of microorganisms to heat generation during BSFL rearing, we simultaneously replicated the experimental setup where we supplemented the substrate with a common preserving agent, potassium sorbate,^22^ and assessed its effect on thermogenesis, microbial activity, and larval development. Potassium sorbate is used as additive (E 202) in food production, and an earlier study has shown that it does not negatively affect larval development in terms of pupal biomass, length, and adult emergence.^7^ We hypothesized that (1) peak temperatures will increase with larval density, (2) density and the supplementation of a preserving agent will influence microbiota composition and larval development, as young larvae rely on microbial assistance for nutrient uptake, and (3) residue properties will differ across larval densities. The combination of measuring rearing performance, high-resolution temperature monitoring, and biomolecular characterization of bacterial communities in substrates and guts provides a comprehensive image of thermogenesis during BSFL rearing.

## Results

### Larval performance and thermogenesis

**Figure 1:**
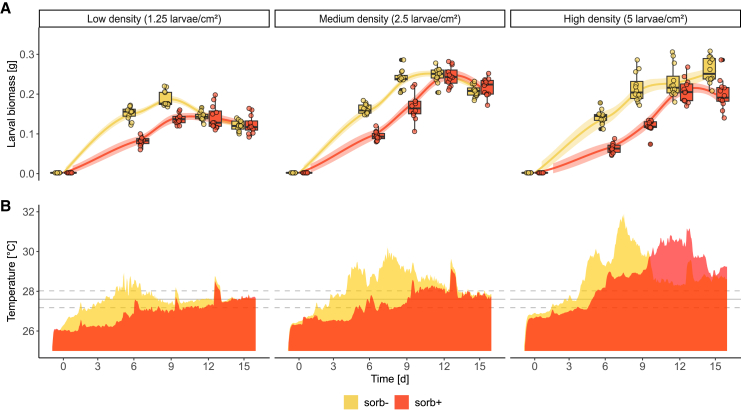
Larval performance and thermogenesis (A) Biomass gain of black soldier fly larvae (BSFL) on food waste containing no (sorb-) or 0.15% (w/w) potassium sorbate (sorb+) (*n* = 4 per treatment). (B) Larval density-dependent substrate temperatures throughout the degradation of food waste by BSFL. The average temperature and standard deviation in the climate room are indicated by the solid and dashed horizontal lines, respectively (*n* = 4 per treatment). Also see Figures S1 and S2.

**Figure 2:**
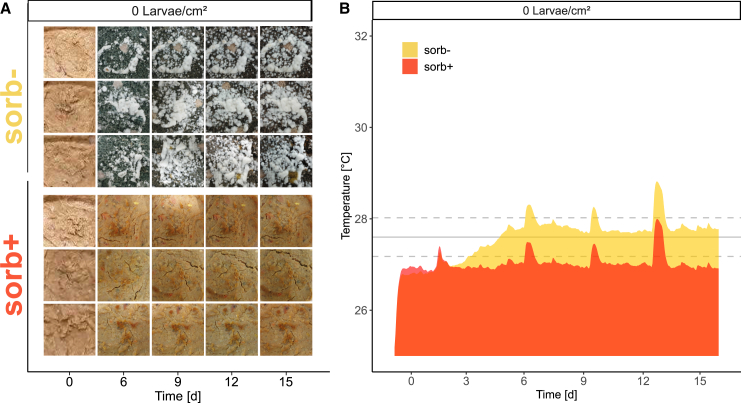
Degradation of food waste and thermogenesis (A) Visual changes in food waste over time containing no (sorb-) or 0.15% potassium sorbate (sorb+). (B) Temperature dynamics in raw food waste without black soldier fly larvae. The average temperature and standard deviation in the climate room are indicated by the solid and dashed horizontal lines, respectively (*n* = 3 per treatment). Also see Figure S2.

The larvae showed primarily density-dependent effects in growth parameters (peak biomass, days to peak, growth rate [GR], and specific GR [SGR]), bioconversion parameters (feed conversion rate [FCR], biowaste conversion efficiency [BCE], waste reduction index [WRI], and substrate reduction [SR]), and thermogenesis, measured as substrate temperatures (Figure 1; Table 1). A treatment-dependent effect was observed in the same parameters except for GR, SGR, FCR, and WRI (Table 1). No significant interaction effect between larval density and substrate treatment was detected. Also, the sorb- and sorb+ groups without larvae differed in their temperature profiles, with sorb- having an up to 1°C higher temperature after three days than sorb+ (Figure 2B). The sorb+ treatment significantly reduced the average peak larval biomass at medium, but not at low and high larval density when compared with the respective control groups (sorb-) (Table 1). It also delayed biomass gain at high density with the highest number of days to reach peak biomass. The highest average biomass in the sorb- control was achieved at a medium density but was not significantly higher than at low or high density (Table 1). At low larval density, the GR and SGR were significantly lower than at medium and high density in sorb- larvae. The delayed growth of sorb+ larvae also had impact on larval thermogenesis, leading to a delayed increase in substrate temperature with later, but insignificantly lower, temperature peaks compared with the sorb- group at each density. Within the control group, the FCR was significantly higher at low density compared to medium and high density. Although less pronounced, similar patterns were observed for the sorb+ treatment. Despite having the lowest FCR, the medium larval density in the sorb- group had the overall highest BCE—more than twice as high as in the low-density treatment of the same group (Table 1). In the sorb+ group, the BCE was highest in the high-density treatment, doubling the bioconversion efficiency of the low-density treatment. The WRI was comparable in various larval densities of the control group. In the sorb+ treatment group, the WRI was comparable at medium to high densities but notably lower at low larval density. A similar trend was observed for the substrate reduction rate.

**Table 1:** Growth and degradation parameters of black soldier fly larvae reared on food waste

Treatment	Density [Larvae/cm^2^]	Peak biomass [mg]	Days to peak biomass [d]	Peak temperature [°C]	Growth rate (GR) [mg/d]	Specific GR (SGR) [%/day]	Feed conversion rate (FCR)	Bioconversion efficiency (BCE) [%]	Waste reduction index (WRI) [g/d]	Substrate reduction (SR) [%]
**sorb-**	**1.25**	187 ± 19^ab^	16^a^	29.4 ± 0.4^ab^	3.4 ± 0.4^c^	41.1 ± 1.1^a^	5.4 ± 0.2^a^	13.8 ± 0.6	7.3 ± 0.3^abc^	73 ± 3^abc^
	**2.5**	240 ± 21^a^	16^a^	31 ± 2.3^ab^	6.2 ± 0.3^ab^	47.1 ± 0.5^b^	2.7 ± 0.1^c^	32.4 ± 0.9	8.5 ± 0.0^a^	85 ± 0^a^
	**5**	230 ± 41^a^	19^ab^	32.3 ± 0.6^b^	6.9 ± 0.9^a^	48.1 ± 1.3^b^	3.0 ± 0.1^bc^	25.2 ± 0.9	7.6 ± 0.3^ab^	76 ± 3^ab^
**sorb+**	**1.25**	141 ± 30^b^	19^ab^	28.6 ± 0.4^a^	3.3 ± 0.6^c^	40.7 ± 1.8^a^	4.0 ± 0.1^ab^	12.2 ± 0.6	4.8 ± 0.1^c^	48 ± 2^c^
	**2.5**	167 ± 33^b^	16^a^	29.2 ± 0.4^ab^	5.7 ± 0.5^ab^	46.3 ± 0.9^ab^	3.4 ± 0.2^abc^	19.4 ± 3.2	6.4 ± 0.8^bc^	64 ± 8^bc^
	**5**	199 ± 38^ab^	22^b^	31.6 ± 0.5^ab^	5.2 ± 0.8^b^	45.2 ± 1.5^ab^	3.0 ± 0.1^bc^	24.8 ± 2.2	7.4 ± 0.6^abc^	74 ± 6^abc^
*Statistics*										
Treatment		*p* = 0.0005∗∗∗	*p* = 0.0432∗	*p* = 0.0435∗	*p* = 0.0822	*p* = 0.1112	*p* = 0.4335	*p* = 0.0735	*p* = 0.0024∗∗	*p* = 0.0023∗∗
		*Df = 1; F* = 17.199	*Df = 1; H* = 4.089	*Df = 1; H* = 4.075	*Df = 1; F* = 3.347	*Df = 1; H* = 2.537	*Df = 1; H* = 0.613	*Df = 1; H* = 3.203	*Df = 1; H* = 9.243	*Df = 1; H* = 9.243
Density		*p* = 0.00527∗∗	*p* = 0.00026∗∗∗	*p* = 0.00009∗∗∗	*p* = 0.00028∗∗∗	*p* = 0.00033∗∗∗	*p* = 0.00043∗∗∗	*p* = 0.00046∗∗∗	*p* = 0.04530∗	*p* = 0.04530∗
		*Df = 1; F* = 9.795	*Df = 2; H* = 16.483	*Df = 2; H* = 21.393	*Df = 1; F* = 19.363	*Df = 2; H* = 16.057	*Df = 2; H* = 15.513	*Df = 2; H* = 15.365	*Df = 2; H* = 6.189	*Df = 2; H* = 6.189
Treatment:Density		*p* = 0.473	*p* = 0.297	*p* = 0.896	*p* = 0.137	*p* = 0.567	*p* = 0.059	*p* = 0.227	*p* = 0.149	*p* = 0.149
		*Df = 1; F* = 0.536	*Df = 2; H* = 2.428	*Df = 2; H* = 0.603	*Df = 1; F* = 2.400	*Df = 2; H* = 1.132	*Df = 2; H* = 5.649	*Df = 2; H* = 2.961	*Df = 2; H* = 3.812	*Df = 2; H* = 3.812

The food waste contained either no (sorb-) or 0.15% potassium sorbate (sorb+) (*n* = 4) and three different larval densities (1.25, 2.5, and 5 larvae/cm^2^). All parameters were calculated based on dry matter biomass and are represented as mean ± standard deviation. To test the main effects as well as interactions of treatment and density, two-way ANOVA was used for normally distributed data (peak biomass, growth rate) and Scheier-Ray-Hare test for non-normally distributed data (days to peak biomass, peak temperature, specific growth rate, feed conversion, bioconversion efficiency, waste reduction index, substrate reduction). Subsequently, Dunn’s posthoc test and Benjamini & Hochberg *p* value correction were used for pairwise comparisons. Superscript lowercase letters indicate statistical differences. Values sharing the same letters indicate no statistically significant difference. Also see Table S2 for the formulas used to calculate these parameters.

### Degradation of food waste and its impact on substrate composition

**Figure 3:**
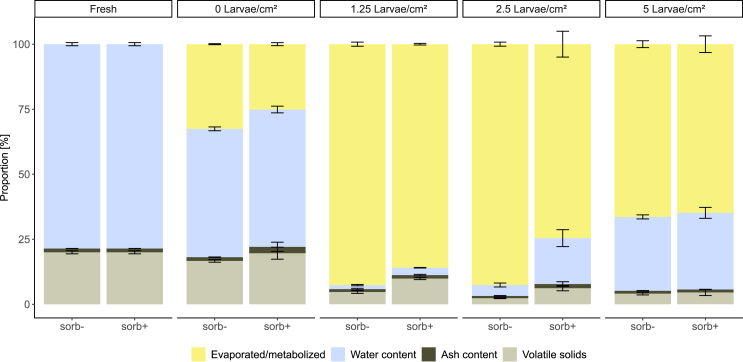
Physicochemical changes in food waste composition Comparison of food waste with no potassium sorbate (sorb-) or with 0.15% (w/w) potassium sorbate (sorb+), in terms of water loss, total solids, and volatile solids before (fresh) and after 15-day degradation by black soldier fly larvae at different larval densities (0, 1.25, 2.5, and 5 larvae/cm^2^; *n* = 4 per treatment, whiskers represent SD). Also see Figures S2 and S3.

The most striking visual difference was observed between the sorb- and sorb+ groups containing no larvae (Figure 2A). While the sorb- control exhibited a dense layer of mold on its surface after six days the latest, no mold growth was observed in the sorb+ treatment throughout the duration of the experiment, indicating the efficacy of the preserving agent. Significant density-dependent effects were observed for all physicochemical parameters measured, whereas significant treatment-dependent effects were observed only for volatile solids reduction and a significant density:treatment interaction was detected only for N content (Table 2). In the sorb+ treatment, substrate temperature remained below the ambient average (Figure 2). The simultaneous peaks in both sorb- and sorb+ treatments were introduced by heat input from body temperature during sampling. Distinct visual changes were observed between the sorb- and sorb+ groups at different larval densities (Figure S2). In both the control and the treatment group, larval activity at the highest density induced the formation of foam on the substrate surface which was not efficiently reduced until the end of the experiment, resulting in the moistest residual substrate across all densities based on the measured water content (Figure 3). Although all treatments received identical feed rations per larva proportionally adapted to the number of larvae at each density, the substrate in the lowest density treatment experienced the highest relative moisture loss. Significant differences in moisture loss between the sorb- and sorb+ groups were only observed at medium density (Table 2).

**Table 2:** Physicochemical parameters of fresh food waste and substrate residues

Treatment	Density [Larvae/cm^2^]	Water loss [%]	Volatile solids reduction [%]	Carbon [%]	Nitrogen [%]	C:N ratio	pH
**sorb-**	**Fresh**	–	–	49.3 ± 0.7^ab^	2.9 ± 0.1^ab^	17.2 ± 0.6^ab^	5.4 ± 0.0^bc^
	**0**	37.2 ± 0.9^ab^	16.5 ± 2.5^ab^	50.0 ± 0.9^a^	3.5 ± 0.5^a^	14.3 ± 1.6^a^	3.9 ± 0.1^abc^
	**1.25**	98.1 ± 0.3^c^	76.4 ± 2.6^abc^	44.0 ± 1.33^ab^	2.6 ± 0.1^ab^	16.8 ± 0.3^ab^	6.6 ± 0.1^a^
	**2.5**	94.7 ± 1.0^bcd^	88.3 ± 0.9^c^	36.6 ± 8.2^ab^	2.0 ± 0.5^b^	18.6 ± 0.8^ab^	7.5 ± 0.1^ab^
	**5**	63.8 ± 1.0^abd^	79.5 ± 2.5^bc^	41.2 ± 2.0^ab^	2.9 ± 0.50^ab^	14.6 ± 2.6^ab^	7.9 ± 0.2^c^
**sorb+**	**Fresh**	–	–	49.3 ± 0.7^ab^	2.9 ± 0.1^ab^	17.2 ± 0.6^ab^	5.4 ± 0.0^bc^
	**0**	32.8 ± 1.6^a^	8.0 ± 6.5^a^	48.9 ± 0.6^ab^	3.1 ± 0.2^ab^	16.1 ± 1.0^ab^	3.6 ± 0.0^abc^
	**1.25**	96.4 ± 0.1^cd^	50.5 ± 2.0^ab^	48.3 ± 1.4^ab^	3.5 ± 0.6^a^	14.2 ± 2.0^ab^	5.7 ± 0.1^a^
	**2.5**	77.5 ± 4.1^abcd^	69.0 ± 5.1^abc^	41.9 ± 6.1^ab^	2.0 ± 0.4^b^	21.2 ± 3.1^ab^	7.3 ± 0.4^abc^
	**5**	62.5 ± 2.7^abd^	77.2 ± 6.0^bc^	42.9 ± 2.8^b^	2.1 ± 0.7^b^	21.5 ± 3.1^b^	7.8 ± 0.2^c^
*Statistics*							
Treatment		*p* = 0.33	*p* = 0.03∗	*p* = 0.42	*p* = 0.68	*p* = 0.34	*p* = 0.63
		*Df = 1; H* = 0.950	*Df = 1; H* = 4.924	*Df = 1; H* = 0.644	*Df = 1; F* = 0.178	*Df = 1; H* = 0.900	*Df = 1; H* = 0.232
Density		*p* = 0.000055∗∗∗	*p* = 0.000361∗∗∗	*p* = 0.000056∗∗∗	*p* = 0.000032∗∗∗	*p* = 0.001342∗∗	*p* = 0.000004∗∗∗
		*Df = 3; H* = 27.148	*Df = 3; H* = 18.416	*Df = 4; H* = 24.785	*Df = 4; F* = 10.179	*Df = 4; H* = 17.812	*Df = 4; H* = 35.174
Treatment:Density		*p* = 0.991	*p* = 0.360	*p* = 0.660	*p* = 0.013∗	*p* = 0.142	*p* = 0.997
		*Df = 3; H* = 0.108	*Df = 3; H* = 3.210	*Df = 4; H* = 2.414	*Df = 4; F* = 3.817	*Df = 4; H* = 6.883	*Df = 4; H* = 0.171

The parameters were measured in substrates both without black soldier fly larvae and with larval densities of 1.25, 2.5, and 5 larvae/cm2 at the beginning and at the end of the experiment. Scheier-Ray-Hare test was used to test the main effects as well as interactions of treatment and density. Subsequently, Dunn’s posthoc test and Benjamini & Hochberg *p* value correction were used for pairwise comparisons. Superscript lowercase letters indicate statistical differences. Values sharing the same letters indicate no statistically significant difference. Also see Figure S3.

The fresh food waste showed a pH of 5.4 (Table 2). In sorb- and sorb+ groups without larvae, the pH decreased to 3.9 and 3.6, respectively. However, with the presence of larvae, the substrate’s final pH increased in accordance with larval density. In substrates with 1.25 larvae/cm^2^, the pH ranged between 5.7 and 6.6, while in those with 5 larvae/cm^2^, it ranged between 7.8 and 7.9 for sorb+ and sorb-, respectively.

Differences were also observed in the substrates’ carbon and nitrogen content and the resulting C:N ratio, although mostly insignificant (Table 2). The C:N ratio increased to 21.5 in the sorb+ treatment at high larval density, while in the sorb- treatment, it ranged from 14.3 to 18.6 (Table 2). The substrate without larvae largely retained its C and N content, while the substrate without preserving agent saw the greatest C and N reduction at medium larval density (Figure S3; Table 2). In the sorb+ treatment, both medium and high larval densities reduced C and N concentrations (Figure S3; Table 2).

### Bacteriobiota in larval guts and food waste

**Figure 4:**
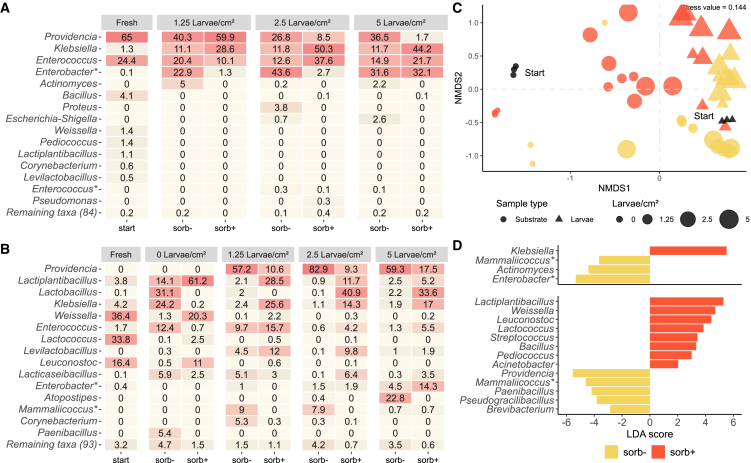
Dynamics of bacterial communities in black soldier fly larvae (A and B) The larvae were reared at various densities (1.25, 2.5, and 5 larvae/cm^2^) on food waste containing no (sorb-) or 0.15% potassium sorbate (sorb+). Heatmaps of the bacteriobiota in (A) larvae and (B) food waste before (“Fresh”) and after bioconversion (0, 1.25, 2.5, and 5 larvae/cm^2^). (C) Non-metric multidimensional scaling of all samples where point shape indicates the sample type, point size indicates larval density, and color indicates the addition of potassium sorbate. (D) Linear discriminant analysis (LDA) of effect size of grouped larval (upper plot) and substrate (lower plot) samples. Genera labeled with asterisks (e.g., “Genus∗”) were assigned by searching for their representative sequences using nucleotide BLAST (Table S1). (*n* = 3 per treatment). Also see Table S1.

The initial bacterial communities in larval guts and substrates strongly diverged (Figure 4C). Guts were dominated by the genera *Providencia*, *Enterococcus*, and *Bacillus* (Figure 4A) accounting for 94% of the identified reads. The dominant bacterial genera in larval guts remained stable across different densities after exposure to food waste, but their relative abundances shifted with the addition of the preserving agent. In the sorb- control, *Enterobacter* ranged between 23 and 44%. In the sorb+ treatment, however, increasing density had a positive effect on *Enterobacter*, increasing its relative abundance in the larval guts from 1% to 3% to 32% at low, medium, and high densities, respectively. The relative abundance of *Providencia* remained largely stable across the different densities in sorb-larvae. However, when exposed to the preserving agent, its relative abundance decreased with increasing larval density, dropping from 60% to 9% to 2%. Notably affected by the preserving agent were the genera of *Klebsiella*, which reached up to 40% higher relative abundance in sorb+ larvae compared with the sorb- control, and *Actinomyces*, which was only detected in sorb- larvae. *Bacillus* sp. made up a higher share of reads in larvae that have not yet been exposed to the substrate and was later only found in very low relative abundances in sorb+ larvae (Figure 4A).

The substrate bacteriobiota were primarily (87%) made up by lactic acid bacteria identified as *Weissella*, *Lactococcus*, and *Leuconostoc* (Figure 4B). The dominant genera in the initial food waste were greatly reduced after larval conversion of the substrate. In the sorb- group containing no larvae, these genera were primarily overtaken by *Lactobacillus*, *Klebsiella*, *Lactiplantibacillus*, and *Enterococcus,* in total accounting for 82% of the reads. In the sorb+ group containing no larvae, the initially dominating genera were reduced to lower relative abundances, with *Lactiplantibacillus* growing from 3.8% to making up nearly two-thirds of the reads (Figure 4B).

PERMANOVA on gut microbial communities showed that the treatment (sorb- vs. sorb+) had the most significant effect on their composition (*F-value*: 11.31, *p* = 0.0009), explaining at least 44% of the variation in the data (R^2^ = 0.44, parOmegaSq = 0.50, for the complementary nature of the two measures see STAR Methods section statistics and data visualization). The effect of various larval densities was also significant (*F-value*: 3.54, *p* = 0.016), explaining at least 14% of the variation (R^2^ = 0.14, parOmegaSq = 0.20). The interaction of treatment:density also resulted in significant differences (*F-value*: 3.78, *p* = 0.009), explaining 15% of the data’s variance (R^2^ = 0.15, parOmegaSq = 0.21).

Both the supplementation of potassium sorbate (*F-value*: 16.96, *p* = 0.0009) and the various larval densities (*F-value*: 9.13, *p* = 0.0009) had significant effect on the substrate bacteriobiota, explaining at least 36% (R^2^ = 0.36, parOmegaSq = 0.56) and 28% (R^2^ = 0.28, parOmegaSq = 0.47) of the variance in the data, respectively. The interaction of treatment:density was also significant (*F-value*: 6.01, *p* = 0.0009) and explained at least 18% of the variance in the data (R^2^ = 0.18, parOmegaSq = 0.36). Larval density and supplementation of potassium sorbate strongly impacted larval gut and substrate bacteriobiota (Figure 4C). LefSe identified *Klebsiella* as characteristic genus in the guts of larvae exposed to the sorb+ treatment, while *Enterobacter*, *Actinomyces*, and *Mammaliicoccus* were overrepresented in the sorb- larvae (Figure 4D). Such overrepresentation was also characteristic of the sorb- control bacteriobiota, along with *Providencia*, *Paenibacillus*, *Pseudogracilibacillus*, and *Brevibacterium*. Moreover, LefSe analysis identified several genera of lactic acid bacteria (*Lactiplantibacillus*, *Weissella*, *Leuconostoc*, *Lactococcus*, *Streptococcus*, and *Pediococcus*) and *Bacillus* as differentially abundant in the sorb+ treatment (Figure 4D).

## Discussion

This study investigated the influence of larval density on thermogenesis and its effects on larval development, microbial ecology, and substrate properties. We hypothesized that higher larval densities would (1) lead to increased substrate temperatures, (2) significantly affecting both larval growth and microbial dynamics, and (3) affect substrate properties. Simultaneously, the experiment was replicated with a preserving agent added to the substrate to reduce microbial contributions to thermogenesis. This research aimed to understand how these density-driven temperature patterns complicate the comparability of experimental results across lab-scale trials, pointing out the need for further research and standardization to enable the translation of findings from small-scale experiments to large-scale applications.

### Larval density drives thermogenesis

Here, we show that even on a laboratory scale, larval density and the resulting thermogenesis significantly impact larval development, microbial ecology, and substrate processing performance (Table 1; Figure 1), stressing that the comparability of experimental outcomes is hampered by varying larval densities. While the original amount of substrate was standardized per larva (Table 3), the differing degradation rates across densities highlight the complexity of these interactions. We measured an increase of up to 2.4°C in peak temperature after doubling the larval density, thus confirming our first hypothesis (Figure 1B). Both treatment and density had a significant effect on temperature peaks; however, no significant interaction was observed, indicating that their effects on temperature peaks are independent of each other (Table 1). The observed temperature peaks at medium larval density align with the findings by Belperio et al.^17^ of peak temperatures of 31.2°C using an omnivorous diet at a larval density of 2.7 larvae/cm^2^. In their study, peaks occurred 11 days post-hatching, while ours occurred at 16 days. They also found that substrate type did not affect substrate temperature, although it was the most influential factor for larval growth. Peguero et al.^19^ also used a density of 2.5 larvae/cm^2^, but the peak temperatures differed greatly depending on the treatment, both in timing (6–12 days after hatching) and intensity (approx. 32.7°C–36.5°C). Few studies on large scale trials continuously measured substrate temperature profiles, and thus, data availability is limited; however, at a similar density of 2.9 larvae/cm^2^ but much larger population size of 8,000 larvae per crate, extreme temperatures of up to 50°C were observed, though no information on larval survival was provided.^18^ Yakti et al.^20^ found that substrate temperatures varied significantly with rearing scale, indicating that larger larval populations generate higher temperatures. Thus, the use of bigger rearing trays containing larger populations of larvae requires more careful monitoring to adjust ventilation and substrate moisture content to the increased risk of excessive heating. Using a population size of 12,000 larvae per crate at a density of 4.5 larvae/cm^2^ (calculated from the data provided in the study), Fuhrmann et al.^23^ recently simulated industrial rearing conditions using food waste and similar feed amounts per larva and day (25 mg vs. 21.4 mg in our study, on dry matter basis). They found that substrate peak temperatures reached approx. 30°C, which aligns with our lab scale findings for a similar density of 5 larvae/cm^2^. Interestingly, the addition of increasing concentrations (2–6%) of a bulking agent not only increased and, in some cases, extended the temperature peaks, reaching approx. 45°C at the highest concentration, but also enhanced CO^2^ production to up to 60 g per h. Loosening organic substrates with bulking agents improved aeration, thereby boosting microbial metabolism and accelerating decomposition^21^; however, bioconversion rate and final larval yield were negatively affected.^23^ Understanding temperature profiles during larval development at specific densities and population sizes is essential for optimizing and managing rearing performance. The industry recognized the importance of thermogenesis as soon as scaling up production became relevant, for example, by adapting cooling, heating, and ventilation to the developmental progress and expected metabolic activity of the larvae (personal observation), whereas academic research is only beginning to address this topic. At large scale facilities, predicting temperature peaks within the substrate could further be exploited to reduce energy consumption needed for maintaining stable rearing temperatures. It was calculated that large scale rearing crates can generate 20–50 Watts of heat during stages of peak temperatures.^23^

**Table 3:** Overview of experimental treatments

Treatment	Larvae/cm^2^	Larvae	Food waste [g]	Layer height [mm]	Substrate volume [cm^3^]	Larvae/cm^3^	Potassium sorbate [g]
sorb-	0	0	451	63	569	0	0
sorb-	1.25	113	113	16	144	0.8	0
sorb-	2.5	226	226	32	289	0.8	0
sorb-	5	451	451	63	569	0.8	0
sorb+	0	0	451	63	569	0	0.676
sorb+	1.25	113	113	16	144	0.8	0.169
sorb+	2.5	226	226	32	289	0.8	0.339
sorb+	5	451	451	63	569	0.8	0.676

Combinations of potassium sorbate supplementation (without potassium sorbate = sorb-, with potassium sorbate = sorb+), larval densities, and details on substrate amounts. All treatments were conducted in semi-transparent plastic boxes (95 × 95 × 95 mm). Also see Figure S1.

### Larval development and thermogenesis are affected by preserving agent

The substrate treatment (sorb- vs. sorb-) had a more pronounced impact on peak larval biomass compared with the larval densities employed, apart from the lowest density (1.25 larvae/cm^2^), at which peak larval biomass was notably reduced. However, as larval density increased, the larvae needed more time to reach their peak biomass, likely due to increased competition for feed. This increased competition is possible despite equal amounts of feed per larva because higher densities can lead to physical interference and environmental stressors such as heat or accumulation of feces, which collectively hinder efficient feed utilization.^15^^,^^24^ In line with this, Guillaume et al.^25^ reported that larval densities between 2 and 6 larvae/cm^2^ resulted in the highest individual fresh and dry mass gain. To account for potential heat generated by microbial decomposition activity—commonly seen in traditional composting, where temperatures can reach up to 82°C^21^—we included a control without larvae to monitor microbial thermogenesis in the raw substrate. Additionally, potassium sorbate was introduced to inhibit molds and yeasts, allowing a more accurate assessment of larval thermogenesis.^22^ The up to 1°C higher temperatures in the sorb- control without larvae likely resulted from increased microbial activity, which was inhibited by sorb+ (Figure 2).^22^ Additionally, unlike the moist surface in the sorb+ treatment, which helped to keep the substrate temperature slightly below the ambient average, the dense mold layer in the sorb- control may have reduced evaporation cooling (Figure 2A). Supplementation of the preserving agent (sorb+) delayed temperature peaks due to slower larval growth but did not significantly reduce them, supporting our second hypothesis that young larvae rely on microbial substrate degradation to aid nutrient uptake. This is backed by observations of Schreven et al.^26^ who found that larvae raised on a sterilized substrate weighed less and developed significantly slower than on an unsterile substrate. Furthermore, larvae hatched from sterile eggs were shown to not grow on autoclaved nutrient media under sterile conditions, which was explained by the lack of essential nutrients that are usually provided by microorganisms.^27^ Previous research has shown that supplementation of yeasts such as *Candida* sp. can increase larval biomass and boosts protein, vitamin B6, and purine metabolism.^28^ The addition of bacterial genera (*Rhodococcus* and *Arthrobacter*) with known biodegrading capabilities has shown similar results by increasing larval growth by up to 22%.^29^ Although the precise mechanisms and extent of microbial contributions to larval development remain largely unexplored, the existing evidence clearly indicates that microbial interactions play a crucial role in larval nutrition, metabolic processes, and overall growth.

### Substrate properties are affected by larval density

Physicochemical features including pH, total and volatile solids, and water and ash content of residual substrates greatly differed across larval densities, confirming our third hypothesis (Table 2; Figure 3). The substrate also decreases in volume due to desiccation and metabolization. Although all treatments were conducted in identical boxes, the lowest larval density registered the highest moisture loss, which can be attributed to increased aeration, reduced heat retention, and greater substrate exposure due to a lower substrate layer height. In turn, the larvae in the high-density treatments encountered the greatest substrate depths (Table 3). Lopez et al.^30^ found that substrate depths greater than 50 mm reduce the BCE, larval survival, and larval biomass as the substrate becomes less accessible. Additionally, they concluded that a high density combined with an increased feeding rate leads to inefficient process conditions. This potentially results in insufficient moisture loss and a wetter residual substrate if the larvae do not process the substrate effectively. Parra Paz et al.^31^ further pointed out that this combination increases leachate production and inhibits the substrate pH from reaching alkaline levels. The desiccation process is crucial as it facilitates separation of the larvae from feeding residues at the end of the process.^32^ Thus, the high residual water content in substrate residues from high density treatments is unfavorable, and in practical applications, the use of drying materials capable of adjusting water content (e.g., wheat bran) would be required to facilitate sieving.^33^ Both pre- and post-consumer food wastes with a water content of 80% were found to induce the fastest larval growth, but only food waste up to a water content of 75% was turned into sievable residues when using a rotary drum reactor for rearing.^34^ Lalander et al.^35^ showed that substrates with high water content inhibit larval bioconversion and survival and can lead to problems with the dry separation of larvae. Active ventilation can assist BSFL in converting substrates with 80–90% water content; however, it has been considered impractical to treat substrates with more than 90% water content, as this would require excessive ventilation and additional processing steps, rendering the bioconversion process impractical.^35^

As expected, in the substrate without larvae, the initial pH of 5.4 dropped to 3.6 due to microbially driven acidification, primarily from the activity of lactic acid bacteria (Table 2).^8^ In contrast, in substrates containing larvae, pH increased with larval density, reaching up to 7.9 due to enrichment of the substrate with larval feces originating from the alkaline hindgut.^36^ Monitoring substrate pH serves as a useful indicator for detecting overfeeding, as excessive feeding rates favor anaerobic conditions, which can disrupt the pH-stabilizing activity of BSFL. In a study using vegetable waste, a feeding rate of 60 mg per larva per day resulted in a substrate pH of 7–8, similar to the levels observed in our medium- and high-density treatments (Table 2).^31^ However, at feeding rates of 200 mg and higher, the pH dropped down to 4–5, indicating unfavorable conditions. Similar to observations made by Lopes et al.,^33^ the substrate C:N ratio was largely unaffected by the treatment across larval densities (Table 2).

So far, studies have explored the impact of different larval densities on development^15^^,^^37^^,^^38^; however, simply reporting larval density as larvae/cm^2^ is insufficient for accurately describing crowding behavior, as larvae tend to cluster in three dimensions within the substrate, and degradation dynamics also differ with population size (i.e., number of larvae per crate).^39^ Thus, we emphasize that in future studies, in addition to larval density, more basic information on the experimental setup, such as crate dimensions, population size, and substrate layer height should be provided to enhance comparability.

### Preserving agent affects gut and substrate bacteriobiota

The composition of bacterial genera dominating larval guts at the beginning and end of the experiment (*Providencia*, *Enterococcus*, and *Klebsiella*) are known as common colonizers of the BSF and its larvae.^40^^,^^41^ Interestingly, *Providencia* and *Enterobacter* exhibited opposing trends in guts of larvae raised on food waste containing potassium sorbate, depending on larval density. As density increased, *Providencia’s* relative abundance dropped, while *Enterobacter* increased (Figure 4A). This indicates that besides treatments and substrates analyzed in studies, density is a highly relevant parameter capable of affecting larval gut microbiota composition. Both genera belong to the phylum of Proteobacteria and class of Gammaproteobacteria, but different families (Morganellaceae and Enterobacteriaceae, respectively), and are widespread colonizers of BSF larval guts.^42^ They have been previously isolated from larval gut environments for potential practical application.^43^ There are indications that *Providencia* is transmitted vertically throughout the insect’s life stages and is assumed to contribute to insect health.^40^^,^^44^ Thus, a significant decrease in relative abundance might be a sign of compromised insect health or disruption of the microbial community balance.

From a substrate perspective, the observed differences in bacteriobiota composition cannot be solely attributed to the effect of potassium sorbate, which primarily targets molds and yeasts.^22^ Additionally, these differences are not consistent across groups without larvae, suggesting that the divergent bacteriobiota patterns in the sorb- and sorb+ groups are more likely due to an interaction between larval density, the preservative, and subsequent changes in substrate physicochemical properties, such as pH and temperature (Figure 4B). However, the supplementation of potassium sorbate as preserving agent strongly promoted the proliferation of lactic acid bacteria (Figure 4D), which are assumed to have beneficial effects also on larval growth.^45^^,^^46^ In contrast, the genus *Paenibacillus*, found indicative of preservative-free food waste, includes *P. thiaminolyticus*, recently identified as the cause of “soft rot” in large-scale facilities, a disease that inhibits larval growth, softens tissue, and leads to death.^47^ However, at least species-level resolution is required to make conclusions about pathogenicity, as for example *Paenibacillus plymyxa* has been previously employed as probiotic for BSF larvae.^48^

### Conclusion and outlook

This study investigated thermogenesis from larval activity as a key factor in feeding trials using food waste with and without potassium sorbate across three larval densities (1.25, 2, and 5 larvae/cm^2^). Density-dependent thermogenesis significantly impacted experimental outcomes, complicating comparability across studies. Temperature-driven effects pose challenges in standardizing rearing conditions, making it difficult to translate lab-scale findings directly to large-scale operations, where heat accumulation and dissipation dynamics differ due to larger substrate volumes, population sizes, requiring better adapted ventilation, heating, and cooling conditions. However, predicting thermogenesis could help reduce operational costs in large-scale rearing by aligning heating and cooling processes with substrate temperature peaks. While reporting larval densities is important, it should be complemented by details on absolute larval numbers, substrate volumes, and layer heights to improve comparability across studies. We therefore advocate for more standardization in this area of research and emphasize that logging substrate temperatures is essential to improve comparability and reproducibility in black soldier fly research. Moreover, the feasibility of our lab-scale findings should be validated in large-scale trials to assess their transferability across different rearing scales and production settings.

### Limitations of the study

This study examined the effects of larval density on thermogenesis and gut and substrate bacteriobiota composition, along with the impact of a preserving agent to elucidate microbial contributions to thermogenesis. While designed at lab scale, findings may have limited transferability to larger-scale studies. Additionally, the use of a single substrate (food waste) is a limitation, as larval development, microbiota, and thermogenesis may vary with substrates of differing physicochemical and structural properties.

## Resource availability

### Lead contact

Further information and requests for resources should be directed to and will be fulfilled by the lead contact, Thomas Klammsteiner (thomas.klammsteiner@uibk.ac.at).

### Materials availability

This study did not generate new unique reagents.

### Data and code availability

• Raw data generated by next-generation sequencing platforms have been deposited at the European Nucleotide Archive (ENA) as bioproject PRJEB67330 (ENA: https://www.ebi.ac.uk/ena/browser/view/PRJEB67330) and are publicly available as of the date of publication.
• All code for statistical analyses has been deposited at https://tklammsteiner.github.io/templarvae and is publicly available at the date of publication.
• Any additional information required to reanalyze the data reported in this paper is available from the lead contact upon request.

## Acknowledgments

We thank Maria Payr and Katharina Stonig for their assistance with laboratory analyses and insect rearing. Moreover, we thank the three anonymous reviewers for their time reviewing this manuscript and their valuable comments and suggestions during the review process. This research was funded in part by the 10.13039/501100002428Austrian Science Fund [grant https://doi.org/10.55776/P35401]. For open access purposes, the author has applied a CC BY public copyright license to any author accepted manuscript version arising from this submission.

## Author contributions

T.K., conceptualization, methodology, software, validation, formal analysis, investigation, resources, data curation, writing - original draft, writing - review and editing, visualization, supervision, project administration, and funding acquisition; C.D.H., methodology, investigation, resources, and writing - review and editing; H.I., resources, writing – review and editing, and funding acquisition; B.C.S.-S., resources, writing - review and editing, and supervision; F.M.S., resources, writing - review and editing, supervision, and funding acquisition.

## Declaration of interests

The authors declare no competing interests.

## STAR★Methods

### Key resources table

**Table:** 

REAGENT or RESOURCE	SOURCE	IDENTIFIER
**Chemicals, peptides, and recombinant proteins**		

Potassium sorbate	Gustav Ehlert GMBH & CO KG	Art.Nr.: 401414

**Critical commercial assays**		

NucleoSpin Soil Kit	Macherey-Nagel	REF 740780.250

**Deposited data**		

Raw data	BioProject	PRJEB67330
Raw experimental data and analyses	Github	https://tklammsteiner.github.io/templarvae

**Experimental models: Organisms/strains**		

*Hermetia illucens* larvae	Laboratory colony of *Hermetia illucens* (Department of Ecology, University of Innsbruck)	N/A

**Software and algorithms**		

R version v.4.3.1	R Core Team	https://www.r-project.org/
R package dada2 v.1.16.0	Callahan et al.^49^	https://benjjneb.github.io/dada2
R package vegan v.2.6.4	Oksanen et al.^50^	https://CRAN.R-project.org/package=vegan
R package MicEco v.0.9.19	Russel^51^	https://github.com/Russel88/MicEco
R package microbiomeMarker v.1.6.0	Yang^52^	https://doi.org/10.1093/bioinformatics/btac438
R package ampvis2 v.2.8.6	Andersen et al.^53^	https://doi.org/10.1101/299537
R package ggplot2 v.3.5.0	Wickham^54^	https://ggplot2.tidyverse.org
SILVA database v.138	Quast et al.^55^	www.arb-silva.de/documentation/release-1381/

### Experimental model and study participant details

#### Black soldier fly lab population

BSFL were obtained from a laboratory colony reared in a ICH750 climate chamber (Memmert, Schwabach, Germany) under steady environmental conditions of 27°C and 60% relative humidity at the Department of Ecology (University of Innsbruck, Innsbruck, Austria). Larvae, pupae, and adults were kept in separate containers, and larval densities were manually controlled. Adults kept in transparent fly cages were exposed to a 16:8 h light:dark photoperiod provided by LED panels (445 × 350 mm; Y51515227, Barthelme, Nuremberg, Germany; described in detail in Heussler et al.^56^) whereas larvae and pupae were kept in opaque plastic containers. The larvae were maintained on a chicken feed (Grünes Legekorn Premium, Unser Lagerhaus, Austria) diet (40:60 w/w mixture with H_2_O) and fed every 2–3 days *ad libitum*. To ensure age equality of the larvae, a batch of eggs harvested on a single day was transferred to an isolated box and supplied with chicken feed for seven days post-hatching.

#### Source and preparation of food waste

The mixed food waste was collected from a local canteen the day before starting the experiment and stored overnight at 4°C. A Vitamix TNC 5200 blender (Vitamix, Cleveland, Ohio, US) was used to homogenize the food waste to a paste-like consistency, with a few smaller chunks (<10 mm) remaining. The food waste was transferred to a food-safe 12 L plastic bucket and thoroughly mixed. Half of the homogenized food waste (5500 g) was left untreated and served as the control group (sorb-). The remaining half was enriched with 0.15% (w/w) potassium sorbate (C_6_H_7_KO_2_), a microbial growth-inhibiting food preservative,^22^ representing the treatment group (sorb+).

#### Feeding trial setup

The boxes were arranged in a randomized order (Figure S1) on a table in the center of an approx. 5-m^2^-sized climate chamber. The ventilation in the chamber was set to a medium level (730 m^3^/h indirect air flow) and an average temperature of 27.5°C. The relative humidity in the air was regulated by a humidifier (Taifun Fog Box IV 8.7 L, GBK, Lüdinghausen, Germany) at 60%. Before adding the larvae, 0.1 g food waste larva^−1^ day^−1^ multiplied by an estimated fattening period of ten days (Table 3) was transferred to the boxes in a single batch-feeding event. The feeding rate was based on personal experience and aligns with that of other similar studies.^57^^,^^58^ The sorb- and sorb+ controls without larvae were filled with the same amount food waste as the highest density treatment (451 g). The substrate moisture was not adjusted throughout the experiment. Based on the average number of neonate larvae counted in 0.5 g (counting repeated five times without replacement), the mass of neonate larvae corresponding to either a low (1.25 larvae/cm^2^), medium (2.5 larvae/cm^2^), or high (5 larvae/cm^2^) density was weighed into each box. Breathable paper tissues mounted on the box openings with rubber bands were used to prevent larvae from escaping, while still allowing the exchange of air. For consistency, control boxes without larvae were covered the same way with paper tissues and treated the same way as the boxes containing larvae. Five randomly selected larvae (repeated three times without replacement) were weighed on Days 0, 6, 9, 12, and 15 to determine average biomass gain. The fresh mass of residues at the end of the experiment was calculated by subtracting the biomass of cleaned larvae (washed with tap water and subsequent dry dabbing) and box tare weight from the total box weight. Samples of substrate and larvae collected at the start and end of the experiment were stored at −20°C until further processing. Growth and degradation parameters, including growth (GR; biomass gain per day, g/d) and specific growth rate (SGR; relative biomass gain day, %/d), feed conversion rate (FCR; feed input per unit biomass gain), biowaste conversion efficiency (BCE; %; Lopes et al.^33^), waste reduction index (WRI; efficiency of waste reduction, g/d), and substrate reduction (SR, decrease in substrate biomass, %), were calculated according to the formulas listed in Table S2.

### Method details

#### Modification of rearing containers

All combinations of treatments (substrate_[sorb-, sorb+]_ × larval density_[0, 1.25, 2.5, 5]_) were set up in four replicate semi-transparent plastic containers (95 × 95 × 95 mm). A hole (Ø = 4 mm) was drilled approximately 10 mm from the ground in each box to fit a single waterproof DS18B20 temperature sensor (AZDelivery, Deggendorf, Germany). The sensors were hot-glued in place to prevent the leakage of fluids from food waste. Nine sensors each were serially connected to four Nano V3.0 Atmega328 CH340 microcontrollers (AZDelivery, Deggendorf, Germany) via a printed circuit board, adding up to a total of 36 temperature sensors. Time and temperature were logged at 1-min intervals and saved to a 32 GB class 10 micro secure digital memory card (Kexin, Shenzhen, China).

#### Physicochemical characterization

The total solids and water contents were determined gravimetrically based on the loss of mass after drying at 105°C for 24 h. The ash content was determined after incineration of the dried samples at 550°C for 5 h, and the volatile solids content was calculated based on the loss on ignition. After the substrate samples were diluted 1:4 with deionized H_2_O and incubated at room temperature for 30 min under regular shaking, pH was measured using a 774 pH meter (Metrohm, Herisau, Switzerland).

#### DNA extraction and 16S rRNA gene sequencing

The substrate and larval samples were thawed overnight at 4°C prior to DNA extraction. From the thawed substrate samples, 250 mg was directly weighed into bead tubes of a NucleoSpin Soil Kit (Macherey-Nagel, Düren, Germany). Buffer SL2 and Enhancer SX were used during the extraction, following the manufacturer’s protocol. Extracts were eluted in 40 μL of Macherey-Nagel elution buffer. DNA concentration and quality were assessed spectrophotometrically (NanoDrop 2000c, Thermo Fisher Scientific, Waltham, MA, US) and by gel electrophoresis. Samples meeting the sequencing requirements were sent for amplicon sequencing on a NovaSeq6000 (Illumina, San Diego, CA, US) platform with a projected sequencing depth of 50k reads per sample.

### Quantification and statistical analysis

#### Sequence data processing and analysis

Raw sequencing data were analyzed using DADA2 v.1.16.0^49^ following the recommended pipeline at https://benjjneb.github.io/dada2/tutorial.html. Briefly, the data were filtered and trimmed using the ‘*filterAndTrim()*’ function, adapting the settings of truncLen = c(200, 200). After estimating the error rates using the ‘*learnError()*’ function, sample inference was performed using the ‘*dada()*’ function. Reads were then merged, and sequences ranging between 251 and 254 bp underwent chimera filtering using the ‘*removeBimeraDenovo()*’ function. Taxonomy was assigned based on the SILVA v.138 database,^55^ and sequences assigned to chloroplasts, mitochondria, or archaea were filtered from the data. Highly abundant ASVs that were not taxonomically identified using the SILVA database in DADA2 were identified via BLAST search (Table S1). The data were then rarefied to the smallest sample size for further analysis.

#### Statistics and data visualization

The Shapiro-Wilk test was used to assess the normal distribution of the data, followed by Levene’s test to evaluate variance homogeneity. For normally distributed data (peak biomass, GR), a two-way ANOVA was performed to assess the main and interaction effects of substrate treatment and density, followed by Tukey’s honest significant difference post hoc test for pairwise comparisons. For non-normally distributed data (Days to peak, Peak temperature, SGR, FCR, WRI, SR, BCE, Total solids, Water, Volatile solids, C, N, C:N, Ash, pH), the Scheirer-Ray-Hare test was used to analyze main and interaction effects, followed by Dunn’s post hoc test for pairwise comparison. Permutational analysis of variance (PERMANOVA) and pairwise PERMANOVA based on Bray-Curtis dissimilarity matrices were calculated using the ‘*adonis2()*’ function in the vegan v.2.6.4^50^ package with permutations = 1000. In addition, partial omega-squared values (parOmegaSq) as unbiased estimators of effect size were calculated using the MicEco v.0.9.19^51^ package. Linear discriminant analysis of effect size (LefSe) was calculated using the ‘run_lefse()’ command in the MicrobiomeMarker v.1.6.0^52^ package. The ampvis2 v.2.8.6^53^ package was used to create relative abundance heatmaps and calculate the non-metric multidimensional scaling based on Bray-Curtis dissimilarity. The figures were created using ggplot2 v.3.5.0.^54^
