# Supplementary material for: Larval density drives thermogenesis and affects microbiota and substrate properties in black soldier fly trials
Source: iScience. 2025 May 30;28(7):112794. doi: 10.1016/j.isci.2025.112794 (PMC12205609; doi:10.1016/j.isci.2025.112794)
Supplement: Document S1. Figures S1–S3 and Tables S1, and S2 [file mmc1.pdf]

**iScience, Volume *28***

# Supplemental information

# Larval density drives thermogenesis and affects microbiota and substrate properties in black soldier fly trials

**Thomas Klammsteiner, Carina D. Heussler, Heribert Insam, Birgit C. Schlick-Steiner, and Florian M. Steiner**

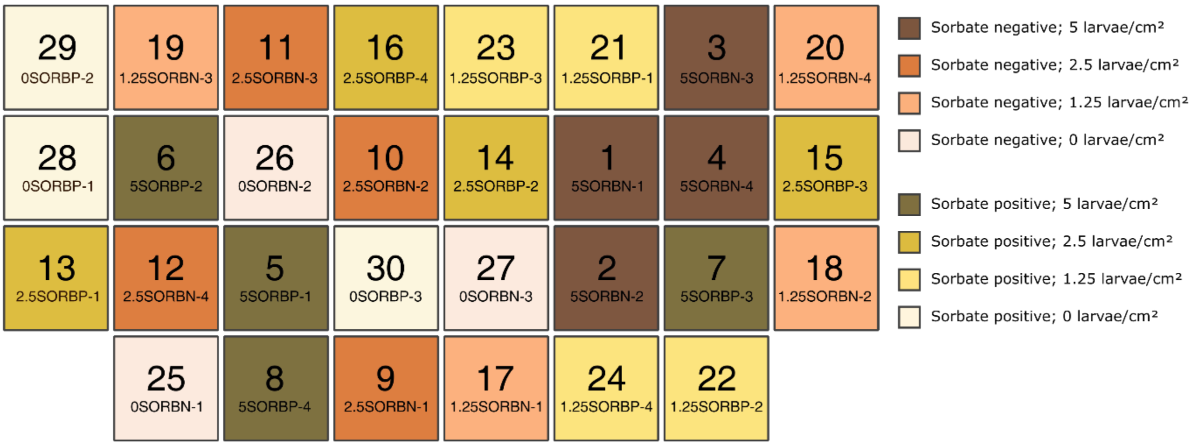


**Figure S1. Randomized set-up of rearing containers defined at the beginning of the experiment.** Related to Figure 1 and Table 3.

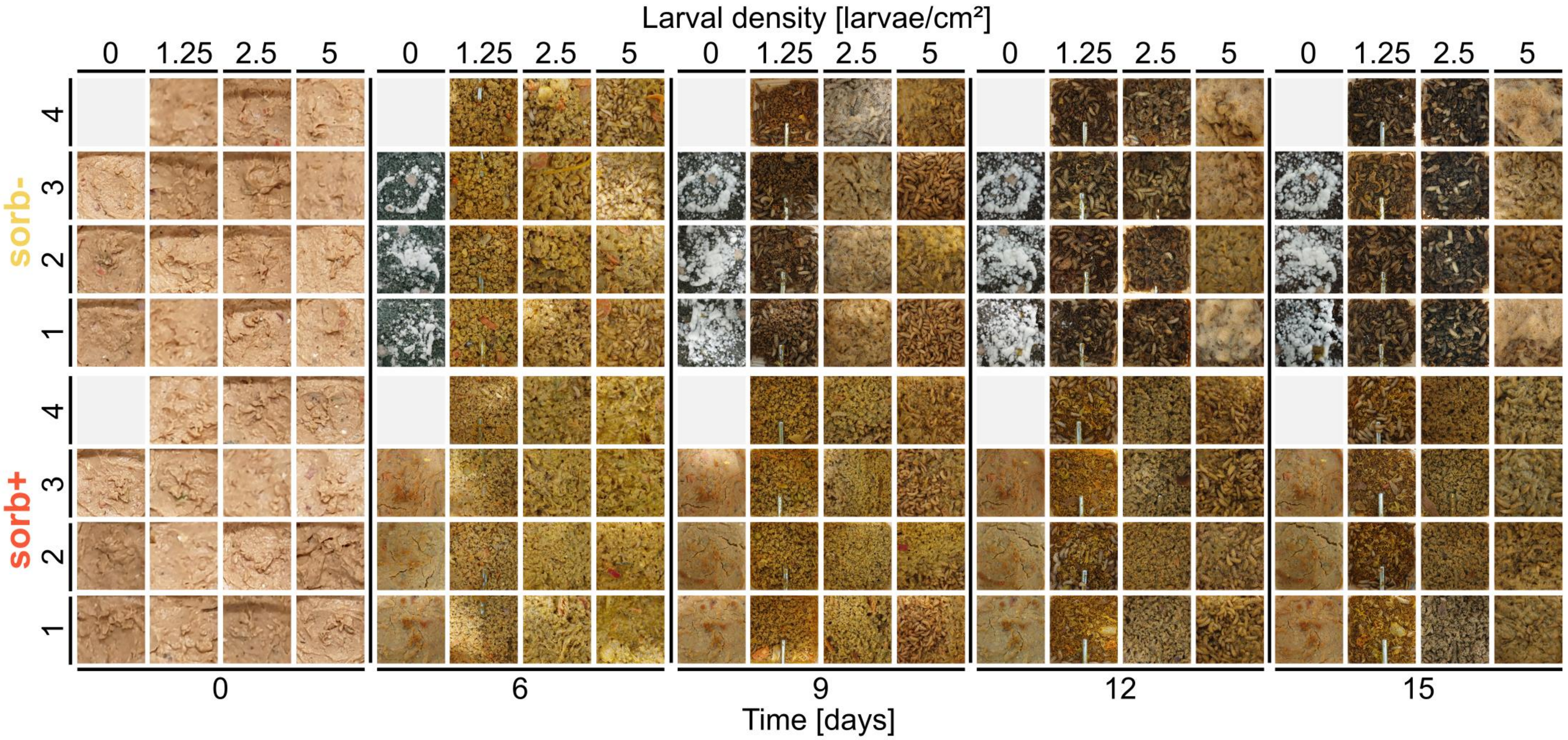


**Figure S2. Changes in the morphology of food waste containing no (sorb-) or 0.15% (w/w) potassium sorbate throughout the experiment (n = 4).** Related to Table 2 and Figure 1,2 and 3.

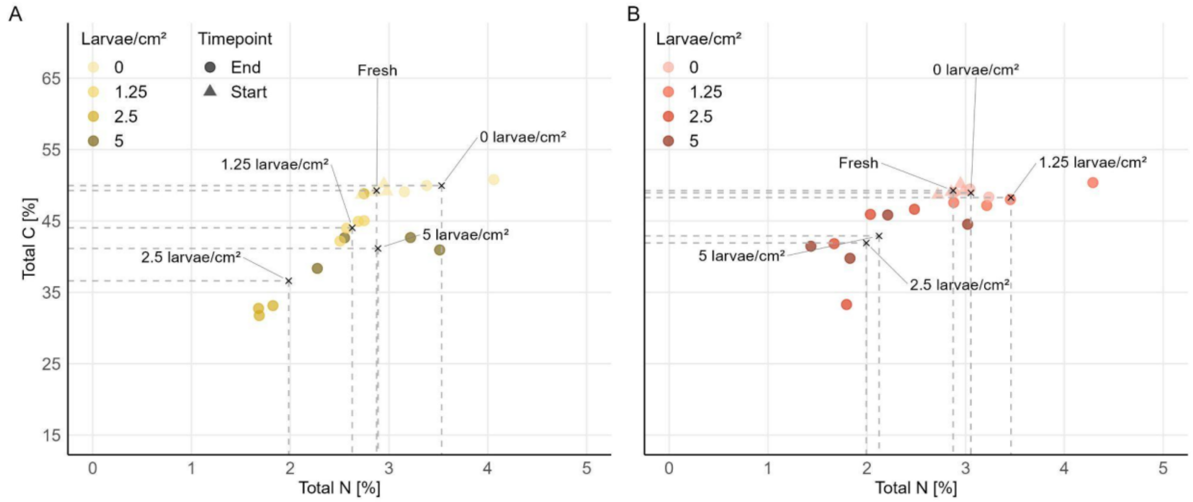


**Figure S3. Variation in total nitrogen (N) and total carbon (C) in food waste.** Food waste containing (**A**) no or (**B**) 0.15% (w/w) potassium sorbate was analyzed before (fresh) after degradation by black soldier fly larvae at larval densities of 0, 1.25, 2.5, and 5 larvae/cm². Cross symbols and corresponding text labels represent the average C:N ratio for each group (n = 4 per treatment). Related to Table 2 and Figure 3.

**Table S1. ASVs identified via BLAST® search.** ASV number, representative sequence, and corresponding nucleotide BLAST® results for highly abundant ASVs that were not taxonomically identified using the SILVA database in DADA2. BLAST results were obtained on 2023-10-11. Related to Figure 4.

| ASV | Representative sequence | Best nucleotide BLAST® result |
|---|---|---|
| ASV3 | TACGGAGGGTGCAAGCGTTAATCGGAATTACTGGGCGTAAAGCGCACGCAGGCGGTCTGTCAAGTCGGATGTGAAATCCCCGGGCTCAACCTGGGAACTGCATTCGAAACTGGCAGGCTGGAGTCTTGTAGAGGGGGGTAGAATTCCAGGTGTAGCGGTGAAATGCGTAGAGATCTGGAGGAATACCGGTGGCGAAGGCGGCCCCCTGGACAAAGACTGACGCTCAGGTGCGAAAGCGTGGGGAGCAAACAGG | NR_102794.2<br>*Enterobacter cloacae*<br>E-value: 9e-132<br>Per. Ident: 100%<br>Acc. length: 1543 |
| ASV13 | TACGTAGGTGGCAAGCGTTATCCGGAATTATTGGGCGTAAAGCGCGCGTAGGCGGTTTCTTAAGTCTGATGTGAAAGCCCACGGCTCAACCGTGGAGGGTCATTGGAAACTGGGAAACTTGAGTGCAGAAGAGGAGAGTGGAATTCCATGTGTAGCGGTGAAATGCGCAGAGATATGGAGGAACACCAGTGGCGAAGGCGGCTCTCTGGTCTGTAACTGACGCTGATGTGCGAAAGCGTGGGGATCAAACAGG | NR_041328.1<br>*Mammaliicoccus sciuri*<br>E-value: 9e-132<br>Per. Ident: 100%<br>Acc. length: 1454 |
| ASV43 | TACGGAGGGTGCAAGCGTTAATCGGAATTACTGGGCGTAAAGCGCACGCAGGCGGTCTGTCAAGTCGGATGTGAAATCCCCGGGCTCAACCTGGGAACTGCATTCGAAACTGGCAGGCTGGAGTCTTGTAGAGGGGGGTAGAATTCCAGGTGTAGCGGTGAAATGCGTAGAGATCTGGAGGAATACCGGTGGCGAAGGCGGCCCCCTGGACAAAGACTGACGCTCAGGTGCGAAAGCGTGGGGAGCAAACAGC | NR_102794.2<br>*Enterobacter cloacae*<br>E-value: 3e-131<br>Per. Ident: 100%<br>Acc. length: 1543 |
| ASV44 | TACGTAGGTGGCAAGCGTTGTCCGGATTTATTGGGCGTAAAGCGAGCGCAGGCGGTTTTTTAAGTCTGATGTGAAAGCCCCCGGCTCAACCGGGGAGGGTCATTGGAAACTGGGAAACTTGAGTGCAGAAGAGGAGAGTGGAATTCCATGTGTAGCGGTGAAATGCGTAGATATATGGAGGAACACCAGTGGCGAAGGCGGCTCTCTGGTCTGTAACTGACGCTGAGGCTCGAAAGCGTGGGGAGCAAACAGG | NR_174226.1<br>*Enterococcus pingfangensis*<br>E-value: 4e-130<br>Per. Ident: 99.60%<br>Acc. length: 1434 |
| ASV54 | TACGTAGGTGGCAAGCGTTGTCCGGAATTATTGGGCGTAAAGCGCGCGCAGGTGGTTTCTTAAGTCTGATGTGAAAGCCCACGGCTCAACCGTGGAGGGTCATTGGAAACTGGGGAACTTGAGTGCAGAAGAGGAAAGTGGAATTCCAAGTGTAGCGGTGAAATGCGTAGAGATTTGGAGGAACACCAGTGGCGAAGGCGACTTTCTGGTCTGTAACTGACACTGAGGCGCGAAAGCGTGGGGAGCGAACAGG | MN931387.1<br>Bacillus litoralis<br>E-value: 3e-127<br>Per. Ident: 100%<br>Acc. length: 733 |
| ASV69 | TACGTAGGTGGCAAGCGTTGTCCGGAATTATTGGGCGTAAAGCGCGCGCAGGCGGCCTTTTAAGTCTGATGTGAAAGCCCACGGCTTAACCGTGGAAGGTCATTGGAAACTGGAAGGCTTGAGGATAGAAGAGGAAAGTGGAATTCCACGTGTAGCGGTGAAATGCGTAGAGATGTGGAGGAACACCAGTGGCGAAGGCGACTTTCTGGTCTATAACTGACGCTGAGGCGCGAAAGCATGGGGAGCAAACAGG | MH291489.1<br>Uncultured bacterium<br>E-value: 3e-127<br>Per. Ident: 100%<br>Acc. length: 429 |

**Table S2. Formulae used to measure growth and waste conversion parameters.** Related to Table 1.

| Parameter | Formula |
|---|---|
| Growth rate S(GR) [g $d^{-1}$] | $GR = \frac{Larva_{final\ biomass} - Larva_{initial\ biomass}}{Time_{days}}$ |
| Specific growth rate (SGR) [%] | $SR = \frac{ln(Larva_{final\ biomass}) - ln(Larva_{initial\ biomass})}{Time_{days}}$ |
| Feed conversion rate (FCR) | $FCR = \frac{Substrate_{input\ biomass} - Substrate_{output\ biomass}}{Larvae_{output\ biomass} - Larvae_{input\ biomass}}$ |
| Biowaste conversion efficiency (BCE) | $BCE = \frac{Larvae_{output\ biomass}}{Substrate_{input}}\ x\ 100$ |
| Waste reduction index (WRI) [g $d^{-1}$] | $D = \frac{Substrate_{input\ biomass} - Substrate_{output\ biomass}}{Substrate_{input\ biomass}}$<br>$WRI = \frac{D}{Time_{days}} \times 100$ |
| Substrate reduction (SR) [%] | $SR = \frac{Substrate_{input\ biomass} - Substrate_{output\ biomass}}{Substrate_{input\ biomass}} \times 100$ |
